# Increased Expression of Zyxin and Its Potential Function in Androgenetic Alopecia

**DOI:** 10.3389/fcell.2020.582282

**Published:** 2021-01-11

**Authors:** Qingmei Liu, Xiangguang Shi, Yue Zhang, Yan Huang, Kai Yang, Yulong Tang, Yanyun Ma, Yuting Zhang, Ji'an Wang, Li Zhang, Qi Zhang, Xiao Liu, Jinran Lin, Jiucun Wang, Wenyu Wu

**Affiliations:** ^1^Department of Dermatology, Huashan Hospital, Fudan University, Shanghai, China; ^2^State Key Laboratory of Genetic Engineering, Collaborative Innovation Center for Genetics and Development, School of Life Sciences, Fudan University, Shanghai, China; ^3^Department of Dermatology, Jing'an District Central Hospital, Shanghai, China; ^4^Human Phenome Institute, Fudan University, Shanghai, China; ^5^Research Unit of Dissecting the Population Genetics and Developing New Technologies for Treatment and Prevention of Skin Phenotypes and Dermatological Diseases (2019RU058), Chinese Academy of Medical Sciences, Beijing, China

**Keywords:** androgenetic alopecia, Zyxin, hair follicle, dermal papilla cell, RNA-seq

## Abstract

Androgenetic alopecia (AGA) is the most common progressive form of hair loss, occurring in more than half of men aged > 50 years. Hair follicle (HF) miniaturization is a feature of AGA, and dermal papillae (DP) play key roles in hair growth and regeneration by regulating follicular cell activity. Previous studies have revealed that adhesion signals are important factors in AGA development. Zyxin (ZYX) is an actin-interacting protein that is essential for cell adhesion and migration. The aim of this research was to investigate the expression and potential role of ZYX in AGA. Real-time polymerase chain reaction (RT-PCR) analysis revealed that ZYX expression was elevated in the affected frontal HF of individuals with AGA compared to unaffected occipital HF. Moreover, increased ZYX expression was also observed within DP using immunofluorescence staining. Our *in vivo* results revealed that ZYX knockout mice showed enhanced hair growth and anagen entry compared to wild-type mice. Reducing ZYX expression in *ex vivo* cultured HFs by siRNA resulted in the enhanced hair shaft production, delayed hair follicle catagen entry, increased the proliferation of dermal papilla cells (DPCs), and upregulated expression of stem cell-related proteins. These results were further validated in cultured DPCs *in vitro*. To further reveal the mechanism by which ZYX contributes to AGA, RNA-seq analysis was conducted to identify gene signatures upon ZYX siRNA treatment in cultured hair follicles. Multiple pathways, including focal adhesion and HIF-1 signaling pathways, were found to be involved. Collectively, we discovered the elevated expression of ZYX in the affected frontal hair follicles of AGA patients and revealed the effects of ZYX downregulation on *in vivo* mice, *ex vivo* hair follicles, and *in vitro* DPC. These findings suggest that ZYX plays important roles in the pathogenesis of AGA and stem cell properties of DPC and may potentially be used as a therapeutic target in AGA.

## Introduction

Androgenic alopecia (AGA) is a common chronic and progressive alopecia disease that can occur in both men and women. The incidence of AGA is higher in Caucasians than in other populations (Otberg et al., 2007). At the same time, the incidence of AGA has a certain age correlation (Severi et al., 2003). Although AGA is not life-threatening, it has a major impact on an individual's appearance and self-confidence and indirectly has a negative impact on the patient's life and social interactions. The main manifestations of AGA in males include frontotemporal hairline recession, whereas females mainly exhibit diffuse hair loss.

The hair cycle mainly consists of anagen, catagen, and telogen phases. In the normal population, the majority of hair is in the anagen phase and lasts the longest. However, in individuals with AGA, the anagen phase is shorter, while the telogen phase is longer. This change leads to the transformation of hair from terminal to vellus and hair follicle miniaturization in individuals with AGA (Lolli et al., 2017). Dermal papilla cells (DPC) exist in hair bulbs and play an important role in inducing hair follicle formation, as well as serve as the main site of androgen activity in hair follicles (Chew et al., 2016; Betriu et al., 2020). DPC can induce hair regeneration and maintain hair in the growth stage through its stem cell characteristics (Yang and Cotsarelis, 2010).

It is well-known that AGA is mainly caused by an increase in androgen levels regulated by genes, particularly dihydrotestosterone (DHT) in the local scalp, which makes the hair follicles smaller. A variety of signaling pathways are involved in the regulation of the hair cycle and hair follicle miniaturization, including Wnt/β-catenin, BMP, and JAK (Vasserot et al., 2019). There are differences in the protein expression level of some markers in the hair frontal parietal region and occipital region. Some inflammation-related markers are only expressed in the areas involved in AGA (Vogt et al., 2017). These differentially expressed genes are involved in several pathways, such as WNT/ β-catenin, androgen, and estrogen (Hochfeld et al., 2019). Differences in these pathways and gene expression levels affect the behavior of hair follicles, including DPC. DPC showed the characteristic of aggregated growth when cultured *in vitro*, which is closely related to the formation of hair follicles and the hair cycle. Previous studies have revealed that adhesion signals are important factors in AGA development. Focal adhesion and cell-to-cell adhesion play major roles in cell aggregation and the growth of DPC, and some extracellular matrices can also affect the aggregation and growth of DPC (Young et al., 2009; Yang et al., 2012).

Zyxin (ZYX), a cytoskeletal protein encoded by *ZYX*, was first described by Mary Beckerle in 1986 as an 82-kDa protein associated with adherent plaques such as focal adhesions (FAs) and actin filaments in cultured chicken embryo fibroblasts (Belgardt et al., 2020). Detailed studies have identified that ZYX is located in FAs and actin stress fibers (Crawford and Beckerle, 1991), and it is structurally and functionally bound to the cytoskeleton (Crawford and Beckerle, 1991). Under stable conditions, ZYX interacts with various integrins, actin-binding, and signaling molecules to affect cell adhesion, movement, and signal transduction. ZYX can also shuffle between the cytoplasm and the nucleus. In the nucleus, ZYX interacts with transcription factors to enhance gene expression (Wang and Gilmore, 2003). Moreover, ZYX constitutes a direct signaling pathway from cytoskeletal-plasma membrane networks to the nucleus, similar to the Wnt/β-catenin pathway and the AP-1 co-activator JAB1 (Wang and Gilmore, 2003). Furthermore, previous studies also revealed that ZYX could mediate the cooperation between Hippo and TGF-β signaling pathways (Ma et al., 2016). The Wnt/β-catenin, Hippo and TGF-β signaling pathways had been implicated in the development of AGA. Therefore, we hypothesize that ZYX plays an important role in AGA.

In the present study, we investigated the expression level and potential role of ZYX on AGA. First, we discovered the upregulated expression of ZYX in the affected frontal HF of AGA patients compared to the unaffected occipital HF. Then, we studied the effect of ZYX downregulation on dorsal hair growth in C57BL/6 mice, cultured hair follicles, and dermal papilla cells. Moreover, we conducted a transcriptome study to delineate the pathways regulated by ZYX. This study provides insights into the molecular mechanism of AGA and may facilitate in the development of novel strategies to control hair loss.

## Materials and Methods

### Assessment of Hair Growth *in vivo*

Wild-type (WT) and ZYX-knockout C57BL/6 mice (6–8 weeks old) were used in the experiment and maintained according to the guidelines approved by the Institutional Animal Care and Use Committee of Fudan University. The dorsal hair of all of the mice was depilated by applying liquid rosin under anesthesia for observation and anagen induction. Baseline and subsequent hair growth were recorded by the same digital camera at a uniform angle and manner for subsequent comparison.

### Human Hair Follicle Organ Dissection and Culture

The scalp hair follicle units of the bald region and non-bald region were obtained from 20 male hair transplant patients with ethical approval and informed consent. The average age of the patients was 30.85 ± 7.31 years, and the average duration of disease was 5.55 ± 2.44 years. According to the Norwood-Hamilton Scale, the degree of hair loss ranged from III V to VI (Table 1). Isolated anagen follicles were used in this study. The isolated human scalp hair follicles were cultured in Williams E medium (Gibco BRL, Grand Island, NY, USA) containing 2 mM glutamine, 10 ng/mL hydrocortisone, 100 U/mL penicillin, and 100 mg/mL streptomycin in 12-well plates at 37.0°C and 5% CO_2_ atmosphere. For the *ex vivo* HF culture, HFs that grew to lengths of 0.3–0.5 mm after 24 h were selected for the subsequent experiment (Kwon et al., 2006).

**Table 1:** Characteristics of all patients in this study.

**Patient No**.	**Age (years)**	**Disease duration (years)**	**Classification (Norwood-Hamilton Scale)**
1	22	3	IVA
2	25	5	IVA
3	27	5	VA
4	34	6	IV
5	28	6	V
6	30	5	V
7	27	5	V
8	28	3	IV
9	29	6	V
10	24	2	V
11	27	3	V
12	33	10	VI
13	47	12	V
14	50	6	IV
15	25	5	III V
16	30	5	IV
17	43	5	IV
18	27	10	VI
19	29	5	IV
20	32	4	III V

### Immunofluorescence Staining

The collected HF samples were embedded in paraffin sections. Then, the sections were sealed with bovine serum albumin and incubated overnight with the primary antibody at 4°C. The antibodies are ZYX (1:500, ab109316; Abcam, United Kingdom) and Ki67 (1:300, ab39012; Abcam). Then, the sections were incubated with a second antibody at room temperature for 1 h. After the second antibody was removed, the nuclei were counterstained with 4-amino-6-diamino-2-phenyl indole (DAPI, Beyotime Biotechnology, China). The stained sections were observed under a microscope (Olympus, Tokyo, Japan), and images were captured.

### Isolation and Culture of hDPCs

DPCs were isolated from the isolated hair follicle bulb and transferred to a polystyrene Petri dish coated with bovine typeI collagen. The characteristics of the DPCs were assessed as shown in Supplementary Figure 1. Penicillin (100 U/mL), streptomycin (100 μg/mL), and 20% heat-inactivated fetal bovine serum were added at 37°C and in a 5% CO_2_ humidified atmosphere and cultured in Dulbecco's modified Eagle's medium. The culture medium was changed every 3 days. The explants were maintained for several days, and when cell growth reached the subfusion state, the cells were harvested in Hank's equilibrium salt solution with 0.25% trypsin/10 mM EDTA and passaged at a ratio of 1:3. Then, the DP cells were cultured in DMEM containing 10% fetal bovine serum, and the third-generation cells were used in the experiment (Moon et al., 2013).

### RNA Isolation, Reverse Transcription, and Real-Time PCR (qPCR)

RNA was extracted from DPCs using TRIzol (Invitrogen), and complementary DNA (cDNA) was synthesized using a high-capacity cDNA reverse transcriptase kit (Application Biosystems). Real-time PCR was performed in a 384-well format with SYBR Green I PCR Kit (Takara, Shiga, Japan) and analyzed in an ABI Prism 7900 detector system (Applied Biological System). The housekeeping gene *GADPH* was used as endogenous control, and the expression of related genes was calculated by the ^ΔΔ^Ct method (Livak and Schmittgen, 2001).

### Cell Proliferation Assay

Cell proliferation was monitored using the xCELLigence system (Baselot in Switzerland and ACEA Biosciences in San Diego, CA, USA). First, the background impedance of E-plate 16 (Roche, East Sussex, UK) was measured using only the growth medium. Then, the 50 μL suspension cells were inoculated into the hole of E-plate 16 and placed in a CO_2_ incubator. Real-time cell analysis (RTCA) software was used to calculate the growth parameters. The cell index was used to represent the percentage of cells in the pore.

### Aggregation Detection of DPCs

First, 6 × 10^4^ cells DPCs were seeded into a 12-plate per well and incubated for 24 h and 72 h for cell attachment. Cells were cultured in the completed DMEM. The aggregation behavior of DPCs was observed under a phase-contrast microscope.

### RNA-seq Data Analysis

One to two micrograms of total RNA extracted from hair follicles was used to prepare sequencing libraries using a TruSeq RNA Prep Kit (Illumina, San Diego, CA, USA), and RNA-seq was performed using a genome analyzer HiSeq X Ten (Illumina). FastQC was used to investigate the sequence quality, and FASTX Toolkit was employed to remove low-quality reads. Kallisto (version 0.44.0) was used for transcriptional quantification without alignment, and tximport was employed to integrate abundance.h5 files to an expressed gene count file, which was used to detect differentially expressed genes (DEGs) using DESeq2 according to the analysis manual. Genes with a *P*-value < 0.05 and |log_2_fold-change| > 1 were determined as DEGs. Kyoto Encyclopedia of Genes and Genomes (KEGG) and Reactome pathway analyses of DEGs were conducted with R Package cluster Profiler (version 3.8.1). The R package ggplot2 (version 3.0.0) was used to draw bar charts, dot plots, and volcano maps, whereas pheatmap (version 1.0.12) was employed to draw a heat map.

### Statistical Analysis

All of the data were analyzed by SPSS 16.0 statistical software, and two independent *t*-tests or one-way ANOVA were used for comparison between groups. Differences with a *P* < 0.05 were deemed to be statistically significant.

## Results

### ZYX Expression Is Upregulated in AGA HFs and Dermal Papilla Cells

**Figure 1:**
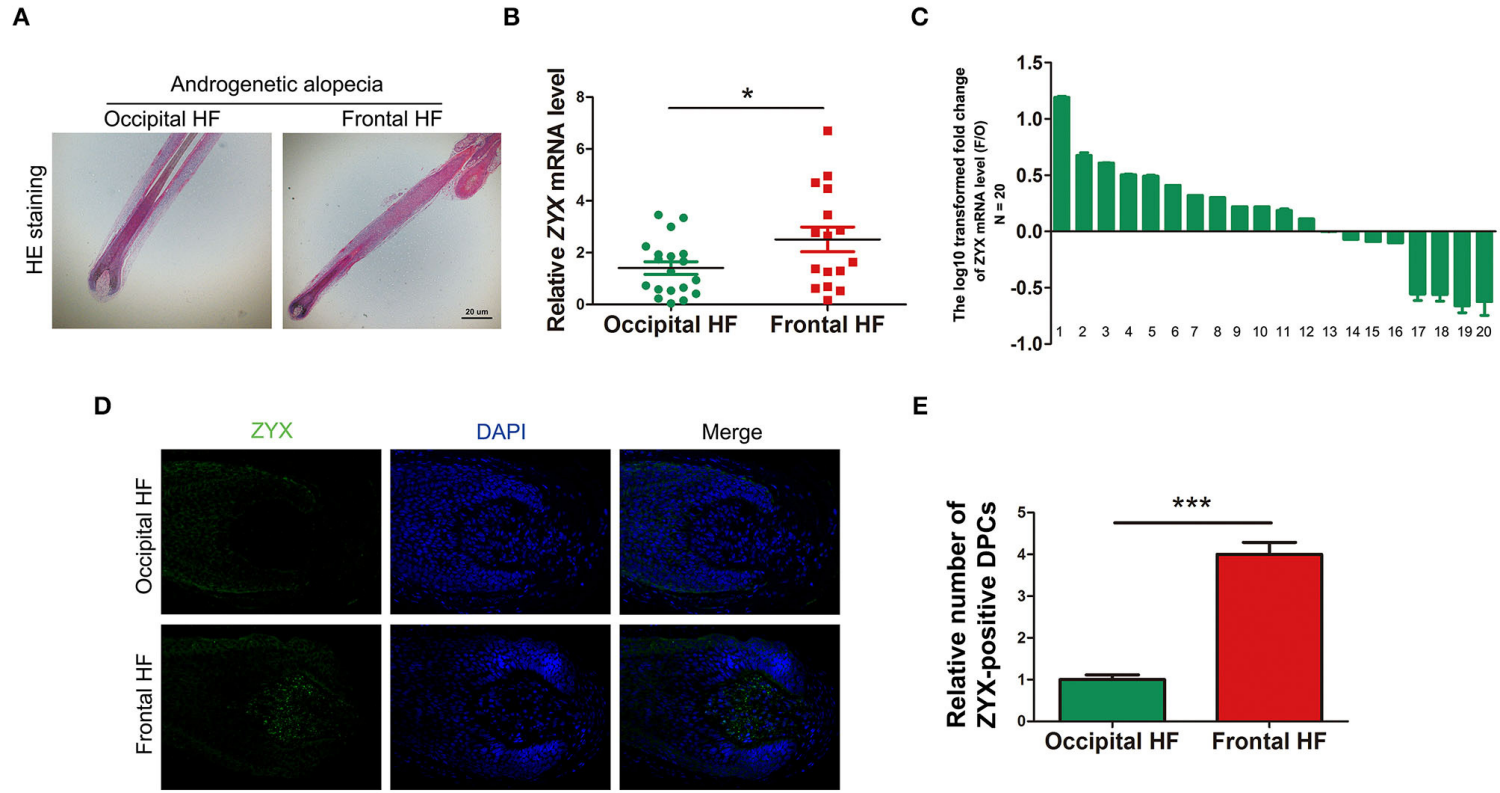
Detection of ZYX in AGA hair follicles and dermal papilla cells. **(A)** HE staining of hair follicles. Scale bar: 5 μm. **(B,C)** mRNA levels of ZYX in hair follicles. *N* = 20. **(D)** Immunofluorescence examination of ZYX in hair follicle tissues. Scale bar: 5 μm. **(E)** Cell counting of ZYX-positive dermal papilla cells. *N* ≥ 3; *P < 0.05, ***P < 0.001 Pregnant group vs. Alendronate group. Control bars represent the mean ± *SD*.

To examine the expression levels of ZYX in AGA, 20 paired HF tissues from AGA patients were collected. HE staining showed that the AGA HFs from occipital nonbald and frontal bald region were anagen HFs (Figure 1A). Then, qPCR analysis was performed and the results showed that the mRNA levels of *ZYX* were significantly higher (~2-fold) in the frontal HF compared to the unaffected occipital HF (Figure 1B). In addition, among the 20 paired tissues from the AGA HF, 12 frontal HFs (60%) showed a significant increase in *ZYX* mRNA expression when compared with paired occipital HFs (Figure 1C). An integrity HF is composed of various cell populations that contribute to the unique biology of HFs during HF formation. Therefore, an immunofluorescence staining assay was used to explore certain cell types that respond to the upregulation of ZYX in AGA HFs. Our results further showed that ZYX was widely expressed in every cell type, but specifically increased in dermal papilla cells (Figure 1D). Additionally, the number of ZYX-positive DP cells increased in the frontal HF group compared with the occipital HF group (Figure 1E). Collectively, we discovered that ZYX expression increased AGA HFs and DPs, which suggests that ZYX play a stimulatory role in AGA.

### ZYX Deletion Promotes Hair Regrowth *in vivo*

**Figure 2:**
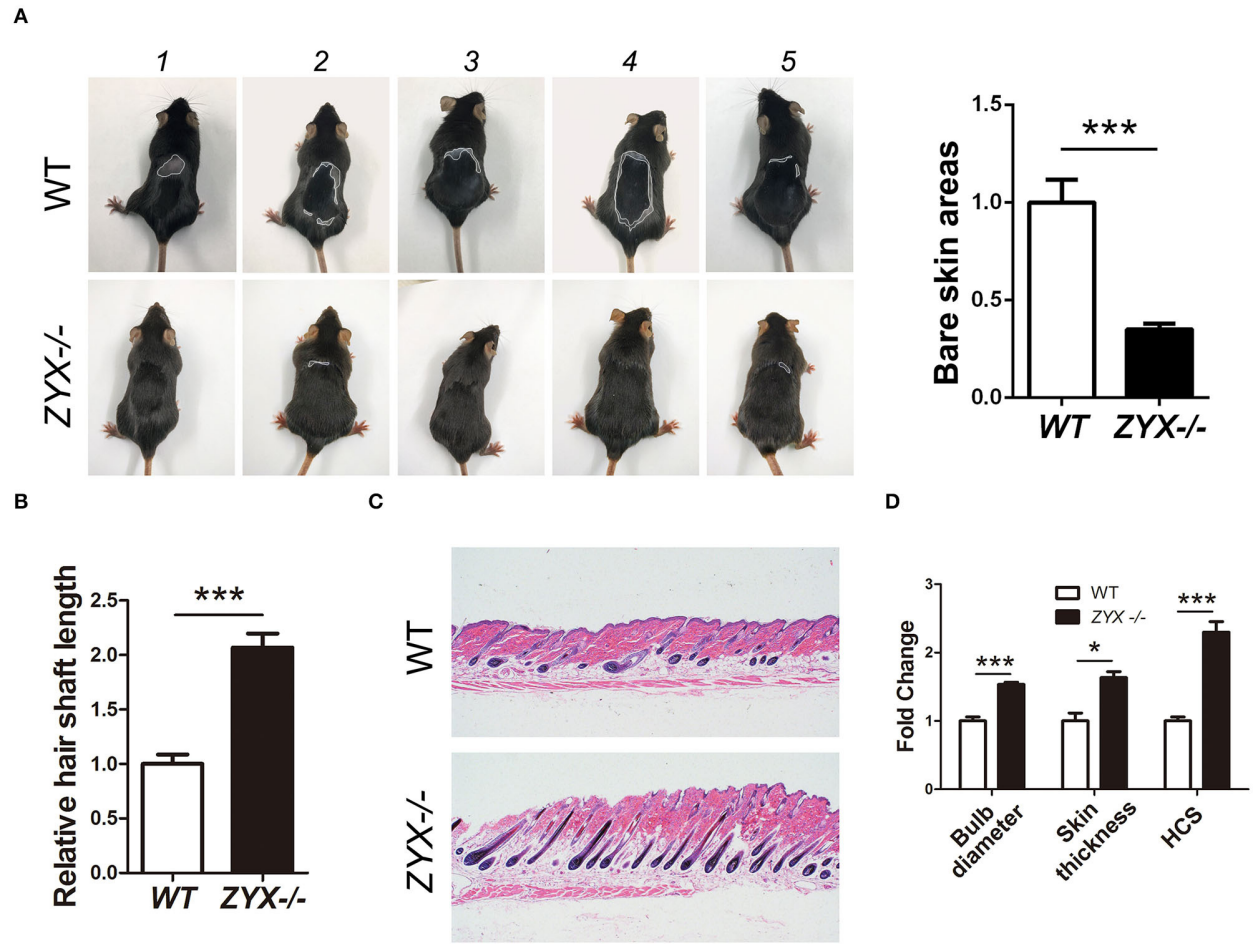
Effect of ZYX on hair growth in mice. **(A)** Size of hair covered skin area in *Zyx-/-* and WT mice. Different morphology of hair shaft **(B)** and hair follicles **(C)** in *Zyx-/-* and WT mice. **(D)** HF percentage and cycling score in *Zyx-/-* and WT mice. Scale bar: 5 μm, *N* =6 per group; *P < 0.05, ***P < 0.001. Control bars represent the mean ± *SD*.

Depilation is an ideal strategy for investigating hair growth. After hair removal, the HF cell cycle was synchronized and accumulated cells in the regrowth phase. To test whether ZYX plays a role in HF cell cycle, WT and *Zyx*-knockout (*Zyx-/-*) mice were depilated. Then, four parameters, namely, length, HF percentage and cycling score (HCS), diameter of hair bulbs, and skin thickness were evaluated to assess HF growth rate. At 24 days after hair removal, photos were taken from five WT and five KO mice; these showed that hair had grown and covered the back skin of the Zyx-/- group, while there were still some bare areas in the WT mice (Figure 2A). The hair shafts were longer and thicker in the *Zyx-/-* mice compared to the WT mice (Figure 2B). We then assessed the morphology of *Zyx-/-*HFs that had thicker skin, and histomorphometric analysis revealed that the subcutis was surrounded by more melanin and bulbs. However, the WT mice showed that most of the hair bulbs were at the dermis subcutis border (Figure 2C). Bulb diameter, skin thickness, and HCS were higher in *Zyx-/-* mice when compared to WT mice (Figure 2D). These results suggest that ZYX deletion facilitates hair regrowth.

### ZYX Knockdown Promotes HF Growth *in vitro*

**Figure 3:**
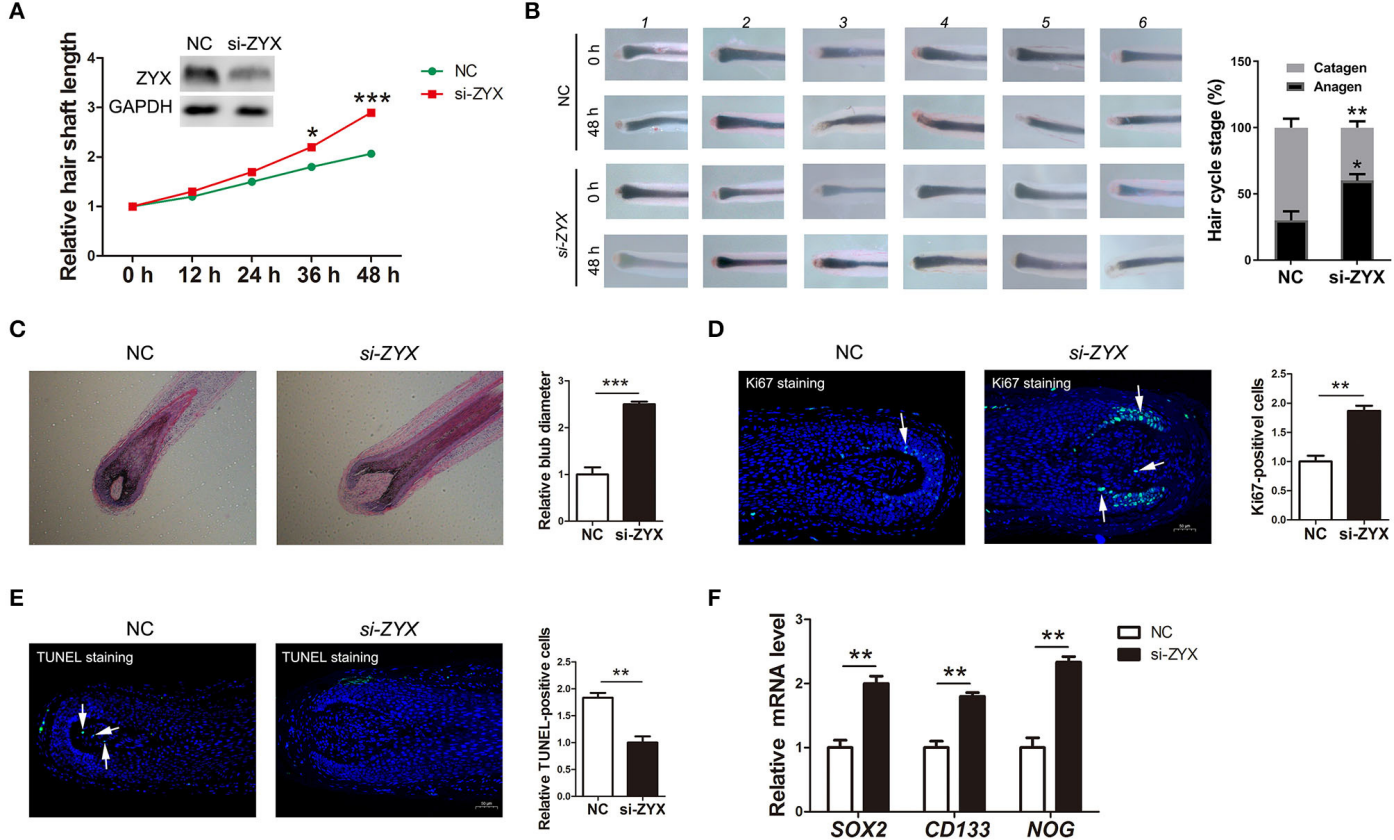
The ratio of hair follicle growth after ZYX knockdown *in vitro*. **(A)** Measurement of the length of hair shaft in ZYX knockdown and negative control groups. **(B)** Distribution of hair follicle cycle in ZYX knockdown and control groups. **(C,D)** Bulb diameter and cell counting of Ki67-positive cells in si-ZYX HFs and NC HFs. **(E)** Evaluation of apoptosis using the TUNEL method after ZYX knockdown. **(F)** Inductivity marker expression in si-ZYX HFs. *P < 0.05, **P < 0.01, ***P < 0.001. Control bars represent the mean ± *SD*.

Cell cycle, apoptosis, and inductivity play important roles in the proliferation and regeneration of HFs. To access whether ZYX is essential for HF development and for phase transition in the HF cycle, human HFs obtained from the AGA patients were treated with *ZYX* siRNA. Histomorphometric analysis showed that ZYX knockdown significantly increased the length of hair shafts compared to the negative control (NC) (Figure 3A). Consistently, 75% of the HFs in the *ZYX* siRNA group was anagen and 25% was catagen, whereas the NC group was 40% anagen and 60% catagen, implying that ZYX deficiency retains the HF cycle in the anagen stage (Figure 3B). Furthermore, HE and immunofluorescence staining revealed larger bulb diameter and increased the number of Ki67-positive cells in *si-ZYX* HFs than that in NC HFs (Figures 3C,D). We next aimed to examine the cell apoptosis effect on *ZYX*-silenced HFs. We then observed that HFs transfected with *si-ZYX* led to a significant reduction in TUNEL-positive cells (Figure 3E). These results suggested that ZYX inhibits HF growth by regulating fibroblast HF cycle and promoting cell apoptosis. To further define the protective role of *si-ZYX* in hair growth, we examined stem cell markers. As expected, si-ZYX significantly induced the expression of SOX2, CD133, and NOG (Figure 3F). Taken together, we concluded that *si-ZYX* promoted HF growth *in vitro* by delaying catagen entry, suppressing cell apoptosis, and sustaining the cell inductivity.

### ZYX Knockdown Sustains the Aggregation Behavior and Facilitates the Proliferation of DP Cells *in vitro*

**Figure 4:**
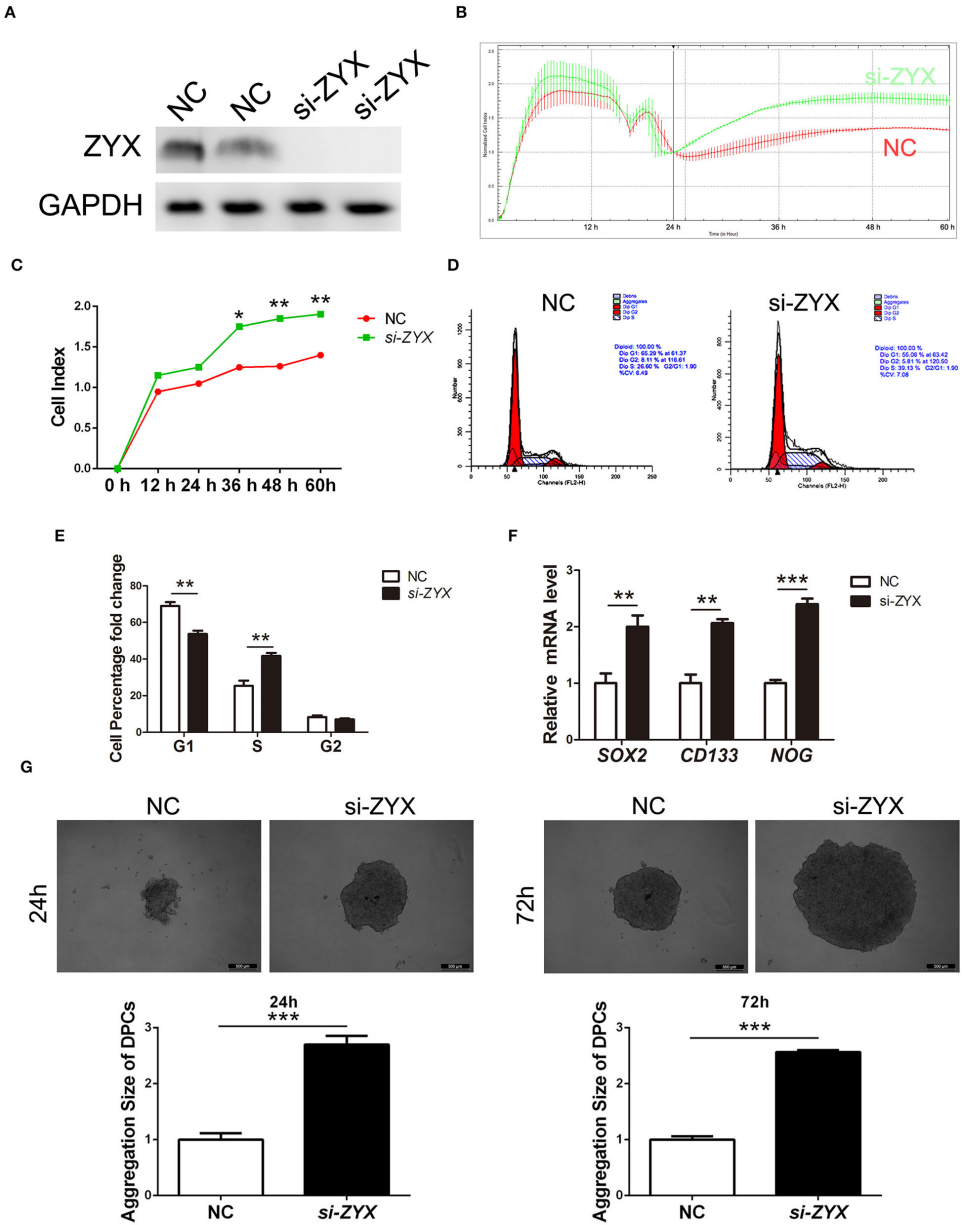
Proliferation ability and inductivity properties in ZYX-deficient DP cells. **(A)** ZYX levels in DPCs after NC or si-ZYX treatment. **(B,C)** RTCA assay of *si-ZYX*- and NC-treated DP cells. **(D,E)** Cell cycle distribution in *si-ZYX*- and NC-treated DP cells. **(F)** Inductivity marker expression in si-ZYX HFs using qPCR assay. **(G)** Inductivity ability assay in si-ZYX or control DPCs. *P < 0.05, **P < 0.01, ***P < 0.001. Control bars represent the mean ± *SD*.

Based on the fact that dermal papilla cells (DPCs) play a critical role in HF growth, formation, inductivity sustainment, and cycling, we investigated the role of ZYX in DP cells. ZYX expression was first knocked down in DPCs by siRNA (Figure 4A). Then, an RTCA assay was performed, which showed that the cell index of DPCs subjected to *si-ZYX* treatment was significantly higher than the NC cells, suggesting that ZYX imparts anti-proliferative effects on DPCs (Figures 4B,C). Accordingly, DPCs treated with *si-ZYX* exhibited an increase in the percentage of cells in the S phase, which was accompanied by a decrease in the percentage of G1 phase cells (Figures 4D,E). Moreover, *si-ZYX* increased the mRNA levels of cell inductivity marker, including SOX2, CD133, and NOG (Figure 4F). Consistently, a cell aggregation assay further revealed that knocking down ZYX facilitated sphere formation (Figure 4G). Taken together, ZYX knockdown accelerated DPC growth by regulating the cell cycle and inducing the aggregation behavior of DPCs.

### Gene Expression Profiles Validate the Growth-Promotional Effect of *si-ZYX* on HFs

**Figure 5:**
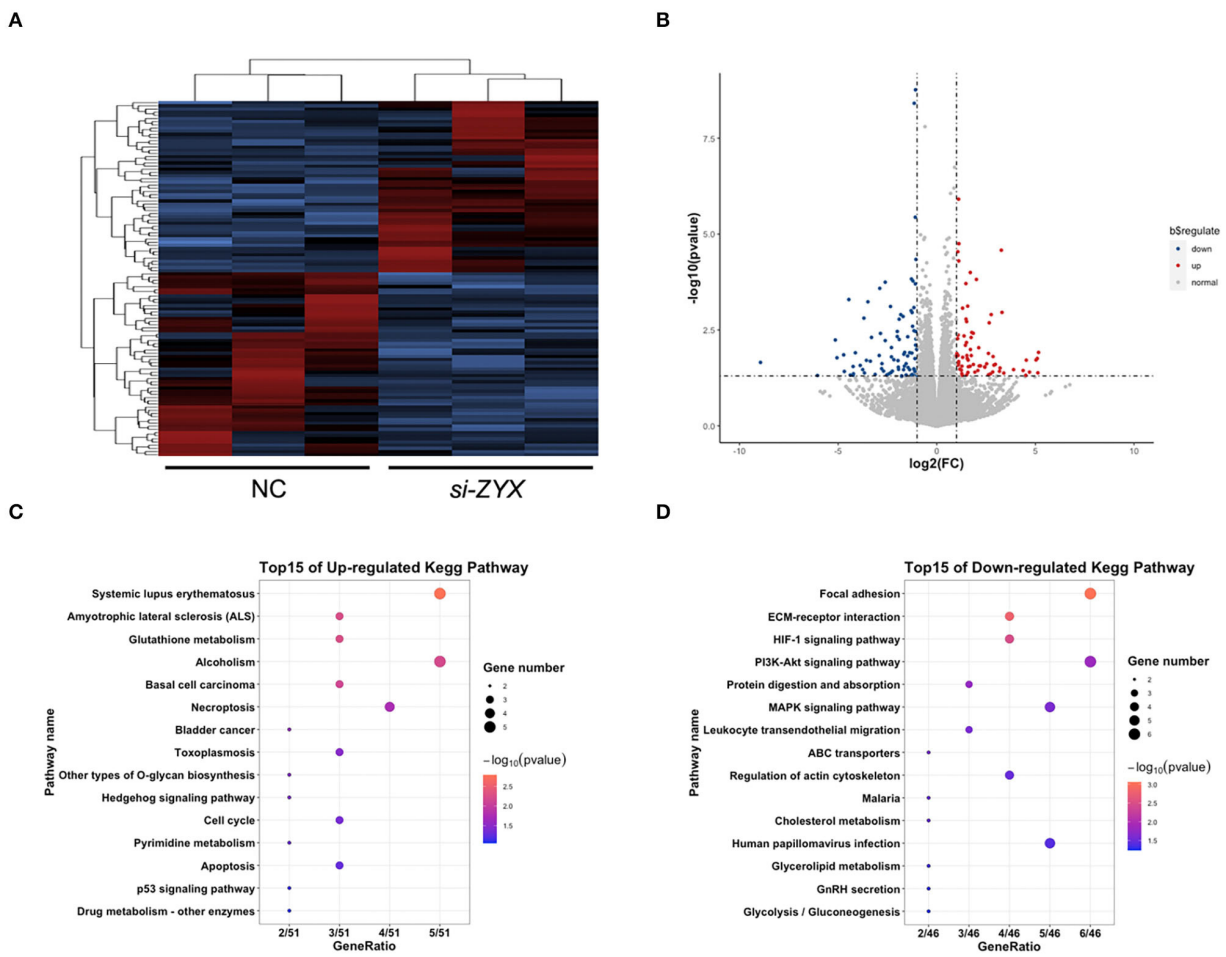
Transcriptomics changes in ZYX-deficient DP cells. **(A)** Heat map of DEGs between ZYX knockdown and control DP cells. **(B)** List of the upregulated and downregulated DEGs. **(C,D)** KEGG analysis of the DEGs. *N* = 3 per group.

To elucidate the potential mechanisms underlying the anti-proliferative role of ZYX in HFs, RNA sequencing (RNA-seq) was performed. Heat map analysis of all of the DEGs based on RNA-seq assay revealed that the gene expression profiles of the NC and ZYX siRNA treatment groups were distinct (Figure 5A). A total of 1,131 DEGs were identified between the two groups, with 576 upregulated DEGs and 555 down-regulated DEGs in *si-ZYX*, respectively (Figure 5B). Further cluster analysis showed that the upregulated DEGs are involved in cell cycle, apoptosis, and other metabolic pathways (e.g., glutathione and pyrimidine), whereas the downregulated DEGs are involved in focal adhesion, HIF-1, PI3K-Akt, MAPK, and other metabolic pathways (e.g., cholesterol and glycolysis) (Figures 5C,D). Taken together, the RNA-Seq data indicated that systemic changes in biopathways were induced by knocking down ZYX.

## Discussion

AGA is the most common form of hair loss in humans, and it is characterized by the miniaturization of hair follicles and gradual hair loss. AGA causes cosmetic problems and psychological distress in affected individuals. The key pathophysiological changes of AGA are limited to HFs in the bald and nonbald regions of the scalp. To date, numerous DEGs and pathways have been identified, including those related to WNT/β-catenin, androgen, hair cycle regulation, hair keratin production, estrogen, melatonin, ephrin, ErbB, and Hippo signaling pathways (Vogt et al., 2017; Hochfeld et al., 2019). An improved understanding of the differences between HF populations and its contribution to AGA pathobiology is thus desirable.

Previous studies have revealed that adhesion signals are important factors in HF and AGA development. For example, α-parvin, which is a FA protein that couples integrins to actin cytoskeleton, is indispensable to HF development (Altstaetter et al., 2020). Furthermore, there is cross-talk between FA/integrin and Wnt signaling pathways, which is one of the most important signaling pathways in AGA (Ridgway et al., 2012). Therefore, we hypothesized that ZYX, an important FA molecule, imparts important effects on HF and AGA development. The present work was designed to study the role of ZYX in AGA. We found that Zyxin is upregulated in the affected frontal HFs of AGA patients compared to the unaffected occipital HFs. To further study the effect of ZYX on HF and AGA, we conducted *in vivo* and *in vitro* studies using mice, cultured HFs, and dermal papilla cells. Using *in vivo* experiments, *ex vivo* cultured HFs and *in vitro* DPCs, we found that reduced ZYX expression enhances hair shaft production, delays HF catagen entry, and enhances DPC proliferation and inductivity. Higher activity of DPCs delays the process of hair miniaturization and promotes shaft elongation. Hair shaft elongation is apparently negatively correlated to hair miniaturization. All of the above results show that ZYX plays an essential role in AGA, and ZYX inhibition might be an effective means for the treatment of AGA and the other hair loss diseases.

DPC stem cell properties play an important role in HF formation and hair growth cycle (Armstrong et al., 2006), and the augmentation of inductivity in DPCs could increase the rate of hair growth and control hair loss. A previous study revealed that the formation of stress fibers and FA complexes contribute to the activity of stem cells (Tucker et al., 2013), which in turn suggests that the FA signaling pathway plays a role in regulating cell inductivity. In the present study, we found that treatment of DPCs with ZYX siRNA significantly promotes cell proliferation. In addition, our real-time PCR analysis and immunocytochemistry staining revealed that ZYX knockdown by siRNA increased the mRNA and protein expression levels of stem cell markers, such as SOX2, CD133, and NOG, in cultured DPCs. Sox-2, a stem cell-related transcription factor, functions in pluripotency maintenance of DPCs. Sox-2 is a key regulator of hair growth that controls progenitor migration by fine-tuning BMP-mediated mesenchymal-epithelial crosstalk (Clavel et al., 2012). Moreover, the presence of stem cell protein markers such as CD133 is indicative of the inductive properties of DPCs. As previously reported, active treatments that induce or enhance stem cell properties of DPCs may be beneficial for hair loss therapeutics. Here, we discovered that ZYX downregulation enhances inductivity maintenance of cultured DPCs. These findings suggest that ZYX plays important roles in HFs and AGA by regulating the activity of DPC. The knowledge gained from our present study may be utilized in the development of novel strategies to control AGA hair loss.

RNA-seq detection was further performed to explore the mechanism of ZYX knockdown in AGA using cultured HFs. KEGG analysis revealed that cell cycle, apoptosis, focal adhesion, ECM-receptor interaction, HIF-1signaling pathway, and PI3K-AKT signaling pathways are the main regulatory pathways that are related to the DEGs detected in ZYX siRNA-treated HFs. This study showed that si-ZYX promoted cultured HF growth, which led to a significant reduction of TUNEL-positive cells in HFs and facilitated DP cell proliferation (Figures 3, 4). These findings also showed that cell cycle and apoptosis pathways are involved in the role of ZYX. Furthermore, RT-PCR was performed to detect changes in the expression level of pathway-related genes. Supplementary Figure 2 shows that the expression levels of PLCB1 (metabolic pathway-related gene), ALDOC/TIMP1/LDHA (HIF-1 signaling pathway-related genes), and ITGB3 (focal adhesion-related gene) were altered after ZYX siRNA treatment. Therefore, we inferred that the effects of ZYX on HF and AGA are also related to FA, HIF-1, and metabolic signaling pathways. ZYX is a classical cytoskeletal protein that is related to FA. Cell adhesion molecules, including cadherins and integrins, are key regulators of stem cell growth and differentiation, for both hair follicle epithelia and epidermal keratinocytes (Akiyama et al., 2000). Integrins and cadherins play a major role in hair follicle growth and proliferation (Silva et al., 2020). A combination of activities simultaneously influences the HIF-1α pathway processes to increase hair density (Juchaux et al., 2020). Minoxidil, an FDA-approved topical drug for AGA treatment, activates an angiogenic pathway HIF-1-VEGF, a potential positive pharmacologic effect for hair growth. Treatment methods against the HIF-1 signaling pathway and hypoxia may be potentially used for the treatment of hair loss. However, we found that the HIF-1 signaling pathway was downregulated by siZYX in this study. Our *in vivo* and *in vitro* data indicated that ZYX plays a role in HF and AGA by regulating the FA signaling pathway via the ITGB1 and HIF-1 signaling pathways, although more research is needed to explore the underlying mechanisms.

## Conclusion

This is the first report that describes the upregulated expression of ZYX in the affected frontal HF of AGA patients compared to the unaffected occipital HF. We found that reduced ZYX expression by siRNA enhances hair shaft production, delays HF catagen entry, and DPC proliferation and inductivity. These findings suggest that ZYX plays important roles in the pathogenesis of AGA and stem cell properties of DPCs, and thus ZYX may be potentially used as a therapeutic target in AGA.

## Data Availability Statement

The original contributions presented in the study are included in the article/Supplementary Material, further inquiries can be directed to the corresponding author/s.

## Ethics Statement

The studies involving human participants were reviewed and approved by Huashan hospital, Fudan University. The patients/participants provided their written informed consent to participate in this study. The animal study was reviewed and approved by Fudan Universtiy.

## Author Contributions

WW, JiuW, and JL conceived and designed the work. QL, XS, YueZ, and YH performed research and collected and analyzed the data. QZ, YT, and YM analyzed the data. KY, Ji'aW, and LZ collected human tissue samples. QL, XS, and YutZ wrote the manuscript. XL modified the manuscript. All authors read and approved the final manuscript.

## Conflict of Interest

The authors declare that the research was conducted in the absence of any commercial or financial relationships that could be construed as a potential conflict of interest.
