# Supplementary material for: Increased Expression of Zyxin and Its Potential Function in Androgenetic Alopecia
Source: Front Cell Dev Biol. 2021 Jan 11;8:582282. doi: 10.3389/fcell.2020.582282 (PMC7829366; doi:10.3389/fcell.2020.582282)
Supplement: Supplementary file 1 [file Data_Sheet_1.docx]

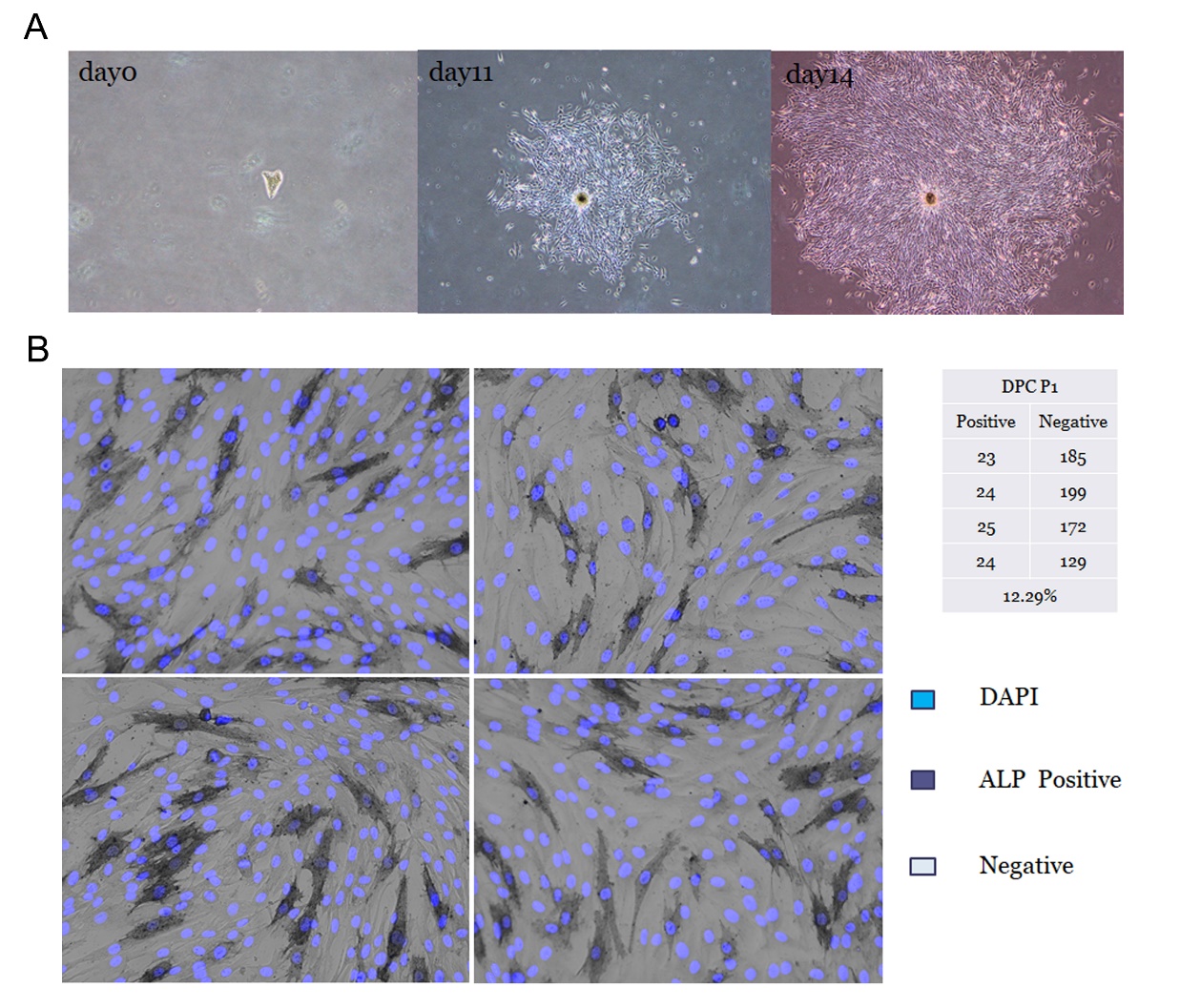


**Supplementary Figure 1. The characteristics of DPCs by morphology and ALP (alkaline phosphatase) detection. (A)** The morphology changes of DPCs from day 0 to day 14. **(B)** The ALP staining of DPC cells.





**Supplementary Figure 2. Validation of DEGs by qPCR analysis.** The mRNA levels of PLCB1, ITGB3, ALDOC, TIMP1 and LDHA in ZYX-deficient DP cells. N = 6, **P* < 0.05, Control bars represent mean ± SD.
